# Transcriptome Identification and Analysis of Fatty Acid Desaturase Gene Expression at Different Temperatures in *Tausonia pullulans* 6A7

**DOI:** 10.3390/microorganisms11122916

**Published:** 2023-12-04

**Authors:** Dianliang Gong, Hua Cong, Shiyu Liu, Liang Zhang, Tianhui Wei, Xinyue Shi, Zhiwei Wang, Xianyao Wu, Jinzhu Song

**Affiliations:** School of Life Science and Technology, Harbin Institute of Technology, Harbin 150006, China; gongdianliang@163.com (D.G.); conghua@hit.edu.cn (H.C.); shiyuliu@hit.edu.cn (S.L.); zltj729@163.com (L.Z.); weitianhui@stu.hit.edu.cn (T.W.); xinyue092300@163.com (X.S.); 22s028013@stu.hit.edu.cn (Z.W.); 18800434243@163.com (X.W.)

**Keywords:** *Tausonia pullulans*, differential genes, *FAD* genes, different temperature modulations, expression analysis

## Abstract

*Tausonia pullulans* 6A7 is a low-temperature yeast strain that can produce lipases. Yeast, which is made up of chassis cells, is an important part of synthetic biology, and the use of the lipase-producing properties of *T. pullulans* 6A7 for the production of fatty acids provides a new pathway for targeted synthesis in yeast cell factories. In this study, we performed RNA-seq on lipase-producing *T. pullulans* 6A7 at different temperatures (15 °C, 20 °C, 20 °C without corn oil, and 25 °C). Therefore, a total of 8455 differentially expressed genes were screened, and 16 of them were *FAD* candidate genes. A Kyoto Encyclopedia of Genes and Genomes (KEGG) pathway analysis of group A (15 °C) vs. group D (25 °C) showed that the pathways of fatty acid biosynthesis (map00061) and the biosynthesis of unsaturated fatty acids (map01040) were significantly enriched. In the proposed temporal analysis of differentially expressed genes among the four temperature modulations, we found differentially expressed genes in nine clusters that had the same expression trends; these genes may be jointly involved in multiple biological processes in *T. pullulans* 6A7. In addition, we found 16 *FAD* candidate genes involved in fatty acid biosynthesis, and the expression of these genes had similar expression in the transcriptome trends with the different temperature treatments. These findings will help in future in-depth studies of the function and molecular mechanisms of these important *FAD* genes involved in fatty acid metabolism in yeast, and they could also be conducive to the establishment of a cellular factory for targeted fatty acid production by using yeast.

## 1. Introduction

Fatty acids (FAs) play an important role in metabolism, stabilizing membrane structure, and mediating the biosynthesis of hormonal signaling molecules. FAs include saturated, monounsaturated, and polyunsaturated fatty acids. Fatty acid desaturases (FADs) are key enzymes in the biosynthesis of lipid metabolism, and they can insert a double bond at a specific location during their involvement in fatty acid metabolism, which is also a process that converts saturated fatty acids into unsaturated fatty acids [1]. Based on their solubility, FADs can be classified into soluble desaturases and membrane-bound desaturases [2]. Studies have shown that fatty acid desaturase genes make up a large gene family with functions that are responsible for the modification and rearrangement of membrane lipids during life activities [3]. The *FAD* gene family can be divided into four subfamilies based on gene function; the first subfamily has one member (*FAD4* gene), the second subfamily has two members (*FAD2* and *FAD6* genes), the third subfamily has three members (*FAD3*, *FAD7*, and *FAD8* genes), and the fourth subfamily has three members (*ADS*, *SLD*, and *DES* genes) [4]. Among the family members, the *FAD2* and *FAD3* genes may be involved in catalyzing lipid desaturation in the endoplasmic reticulum (ER), and the *FAD4*, *FAD6*, *FAD7*, and *FAD8* genes may be involved in catalyzing lipid desaturation [4,5,6].

*Tausonia pullulans* has been reported to be a low-temperature yeast species that can produce lipases [7]. Lipases mainly come from bacteria, fungi, and yeast [8]. When used as cell factories, yeasts are the world’s leading source of biotechnological products, outproducing other industrial microorganisms; their uses include the production of certain products or enzymes in cell factories, such as lipases [7], steroids [9], statins [10], polyamines and polyamine analogs [11], high-value phytochemicals [12], natural products [13], etc. Lipase enzymes have applications with biotechnological and industrial potential when obtained by using yeast species [14]. Lipase-producing species of commercial importance include *Tausonia pullulans*, *Candida rugosa*, *Candida antarctica*, *Yarrowia lipolytica*, and other species of *Pichia* sp., *Rhodotorula* sp. and *Trichosporon* sp. [7,8,15,16]. Some of the genes for lipases in these strains have been cloned or overexpressed for lipase biosynthesis, but the involvement of *FAD* genes in fatty acid synthesis has not been studied in the yeast of *Tausonia pullulans* 6A7.

In view of the promising applications of lipase production in yeast, the screening and identification of functional genes involved in fatty-acid-biosynthesis-related functions in yeast are particularly important. In this study, RNA-seq technology was used to sequence the transcriptome of lipase-producing *Tausonia pullulans* 6A7 with different temperature modulations (15 °C, 20 °C, 20 °C without corn oil, and 25 °C). Meanwhile, by using GO functional annotation, KEGG analysis, and a proposed temporal analysis, we screened the differentially expressed genes at different temperatures and obtained the *FAD* candidate genes involved from *T. pullulans* 6A7. These provide references for the directed production of lipase in yeast cell factories and facilitate the establishment of a synthetic biological gene library of candidate genes in yeast as chassis cells, which also can provide theoretical foundations and genetic resources for cell factories.

## 2. Materials and Methods

### 2.1. Collection and Secretion of the Lipase-Producing Microorganism Tausonia pullulans 6A7

During a study of low-temperature yeast diversity in Beiji Village, Mohe City, Heilongjiang Province, the 6A7 strain was found to have good lipase-producing activity according to ITS and 26S, and it showed 100% similarity to the low-temperature yeast *Tausonia pullulans*; therefore, it was named *Tausonia pullulans* 6A7. Previous studies have shown the lipase-producing ability of *Tausonia pullulans*, but its ability to produce lipase at low temperatures has not yet been studied in depth. A ring was picked from the slant medium, inoculated into a 100 mL triangular flask containing 40 mL of YM culture medium, and incubated at 20° for 24 h, at which time the OD600 was about 0.8, and then, inoculated into YM culture medium for incubation at different temperatures for 5 days. The sample of *Tausonia pullulans* 6A7 underwent four treatments, namely, 15 °C (A), 20 °C (B), 20 °C without corn oil (C), and 25 °C (D). Each sample had three biological replicates that were stored in a in a deep-freezer at −80 °C for the subsequent experiments.

### 2.2. RNA Extraction, Detection, and RNA-Seq in Tausonia pullulans 6A7

Total RNA was extracted from the samples described in Section 2.1, and the concentration and purity of the extracted RNA were examined by using a Nanodrop2000 (Thermo Fisher Scientific Ltd., Waltham, MA, USA). The integrity of the RNA was detected by using agarose gel electrophoresis, and the RIN value was determined using an Agilent2100 (Agilent Technologies Inc., Santa Clara, CA, USA) [17]. A single-construction library required total RNA ≥ 1 ug and OD260/280 ≥ 1.8. Sequencing experiments were performed by using the Illumina Truseq (TM) RNA sample prep kit method for library construction, and RNA-seq was conducted using the Illumina Novaseq 6000 sequencing platform (Illumina, San Diego, CA, USA) [18].

### 2.3. Analysis of Differentially Expressed Genes (DEGs) in Tausonia pullulans 6A7 at Different Temperatures

In this study, an analysis of differentially expressed genes (DEGs) in *Tausonia pullulans* 6A7 at different temperatures was performed by using the DESeq2 package [19] in R language version 4.2.2. The analysis was based on expression data, and all of the DEGs were determined based on a false discovery rate (FDR) of <0.05 and a fold change of ≥2, both of which were met simultaneously. In order to study the expression patterns of the DEGs at different temperatures, a heat map of the expression patterns was drawn by using TBtools version 1.130 [20].

### 2.4. GO Functional Enrichment Analysis of Differentially Expressed Genes in Tausonia pullulans 6A7 at Different Temperatures

To better study the functions of differentially expressed genes in *Tausonia pullulans* 6A7 at four temperatures, Blast2go version 6.0.3 was used for GO functional annotation of the differentially expressed genes [21], and the DEGs were annotated and divided into three GO classes, namely, molecular functions (MFs), cellular components (CCs), and biological processes (BPs). At the same time, Goatools software (https://github.com/tanghaibao/GOatools, accessed on 3 May 2023) was used for a GO enrichment analysis of the DEGs in the gene set [22]; such analyses are usually corrected for *p*-values by default, and when the correction was *p* < 0.01, the GO function was considered to be significantly enriched.

### 2.5. KEGG Enrichment Analysis of Differentially Expressed Genes in Tausonia pullulans 6A7 at Different Temperatures

In order to investigate the involvement of differentially expressed genes in the biosynthetic pathway in *Tausonia pullulans* 6A7 at different temperatures, the Kyoto Encyclopedia of Genes and Genomes (KEGG) (http://www.genome.jp/kegg/, accessed on 16 May 2023) was used for analysis of their KEGG pathways, and hypergeometric tests were applied to identify differentially expressed genes in specific pathways that were significantly enriched by using scripts written in R language (version 4.2.2). The calculation principle was the same as that in the GO functional enrichment analysis, and by default, when *p* < 0.05, the KEGG pathway was considered to be significantly enriched.

### 2.6. Proposed Temporal Analysis of Differentially Expressed Genes in Tausonia pullulans 6A7

A temporal analysis of the DEGs was conducted by using the TC-seq packages in R language. The expression data of differentially expressed genes were used to study their temporality. A statistical analysis of the number of differentially expressed genes in different clusters was performed by using GraphPad Prism version 8.3.

### 2.7. Screening of FAD Candidate Genes in Tausonia pullulans 6A7

To ensure the completeness and accuracy of the identification of *FAD* genes, we used the results of the KEGG enrichment analysis and GO annotation analysis to screen the *FAD* candidate genes in *Tausonia pullulans* 6A7 at different temperatures. At the same time, a dynamic radar map of the candidate genes’ expression at different temperatures was constructed so as to determine their expression patterns and identify the candidate genes.

### 2.8. Fluorescent Quantitative RT-PCR Analysis of FAD Candidate Genes in Tausonia pullulans 6A7 at Different Temperatures

Based on the results of the KEGG enrichment analysis and GO annotation analysis, we screened and obtained the *FAD* candidate genes and performed a fluorescent quantitative RT-PCR analysis on them. The *NADPH* gene was selected as the reference gene. Reduced nicotinamide adenine dinucleotide (NADPH) is a key influencer in fatty acid synthesis. The *NADPH* gene as the reference gene can be expressed at a constant level, independent of temperature. HiScript III RT SuperMix for qPCR (+gDNA wiper) (Nanjing Vazyme Ltd., Nanjing, China) was used for reverse transcription, and the experiments were performed according to the product’s instructions. In this study, the following qRT-PCR reactions were used: predenaturation at 95 °C for 180 s; qRT-PCR at 95 °C for 10 s and 60 °C for 30 s for 40 cycles; melting at 95 °C for 5 s, 65 °C for 60 s, 97 °C for the next period, and 40 °C for 30 s. A relative quantitative analysis of the data was performed by using the Roche Light Cycler 480 (Roche Ltd., Basel, Switzerland) fluorescent quantitative PCR instrument with the 2^−ΔΔCT^ method.

## 3. Results

### 3.1. Screening of Differentially Expressed Genes in Tausonia pullulans 6A7 at Different Temperatures

**Figure 1:**
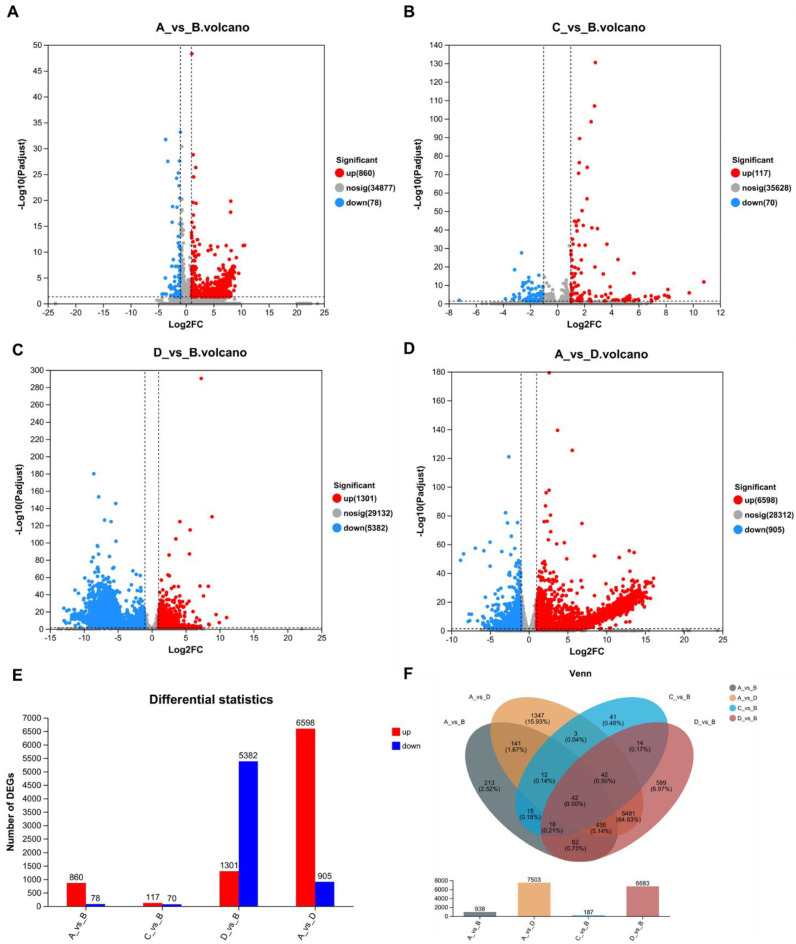
Volcano map and Venn diagram of differentially expressed genes in *Tausonia pullulans* 6A7 at different temperatures. (**A**) Volcano plot of A vs. B. (**B**) Volcano plot of B vs. C. (**C**) Volcano plot of B vs. D. (**D**) Volcano plot of A vs. D. (**E**) Statistical map of the differentially expressed genes. Red indicates upregulated genes, and blue indicates downregulated genes. (**F**) Venn diagram of differentially expressed genes between groups at different temperatures. These genes were identified as DEGs when the FDR was <0.05 and the fold change was ≥2.

In our study, we conducted an in-depth examination of transcripts from *Tausonia pullulans* 6A7 at different temperatures obtained through RNA-seq. They were grouped according to four different temperature points, namely, 15 °C (A), 20 °C (B), 20 °C without corn oil (C), and 25 °C (D); then, DEseq2 was used to analyze the expression of DEGs in different groups. As a result, we found that there were 938 differentially expressed genes between A and B, of which 860 were upregulated genes and 78 were downregulated genes (Figure 1A,E). Between C and B, there were 187 differentially expressed genes, of which 117 were upregulated genes and 70 were downregulated genes (Figure 1B,E). Between D and B, there were 6683 differentially expressed genes, of which 1301 were upregulated genes and 5382 were downregulated genes (Figure 1C,E). Between A and D, there were 7503 genes, of which 6598 were upregulated genes and 905 were downregulated genes (Figure 1D,E).

**Figure 2:**
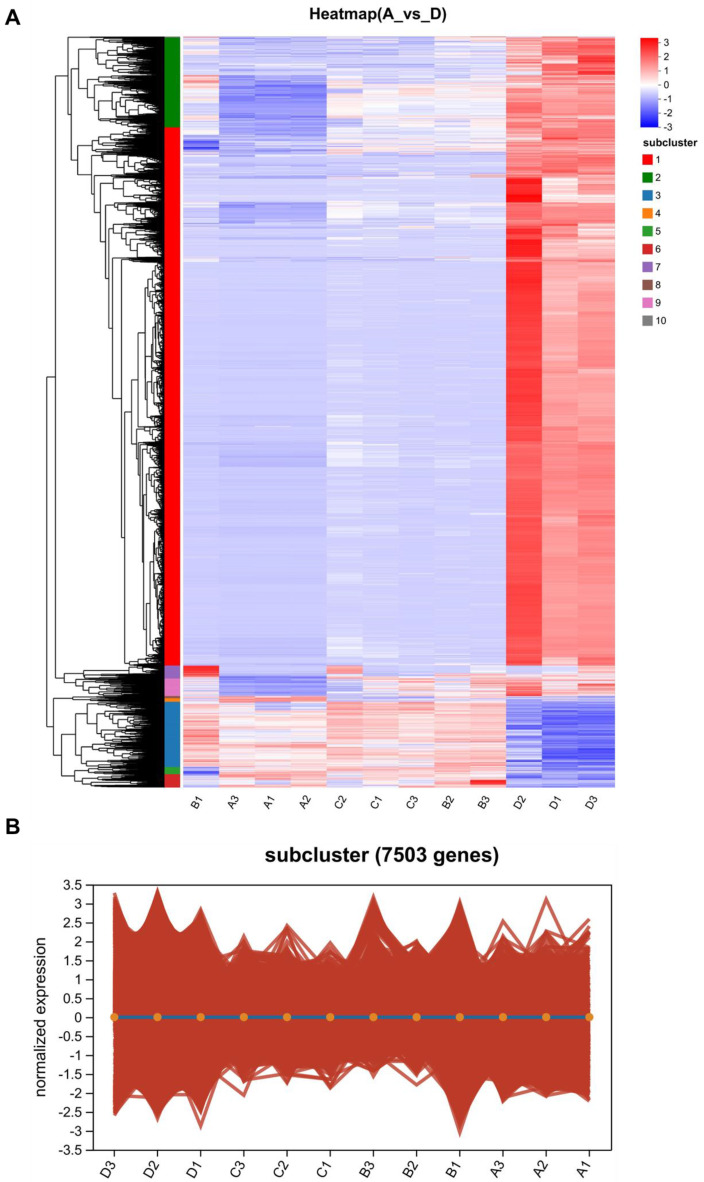
The expression patterns of DEGs in *Tausonia pullulans* 6A7 between temperatures A and D. (**A**) Heatmap of the differential gene expression patterns in 6A7 between two temperatures. A is 15 °C, and D is 25 °C. (**B**) Subcluster trend map of differential gene expression patterns between temperatures A and D. The x-axis indicates the samples at different temperatures. The y-axis indicates the logarithmic value of the differential expression of the genes in that sample. Each line (red) in the graph represents the trend of the change in one gene, and the fitted line (blue) represents the trend of the mean expression of all genes in that subcluster.

By merging the data on the DEGs among the different groups, a transcriptome database with a total of 8455 differentially expressed genes was successfully constructed. A Venn diagram analysis was performed, and it was shown that, among the different subgroups, 42 genes were significantly differentially expressed at the four temperatures, 507 genes were significantly differentially expressed at three temperatures, 5716 genes were significantly differentially expressed at two temperatures, and 2190 were significantly differentially expressed at only one temperature (Figure 1F). The greatest number of differentially expressed genes were present between groups A and D (15 °C and 25 °C); this finding helped in screening for important candidate genes that were differentially expressed between high and low temperatures in *Tausonia pullulans* 6A7. Finally, a heat map of the DEGs between A and D is plotted in Figure 2; we found that the DEGs had different modes of presentation at different temperatures (15 °C and 25 °C), and their expression varied in different subclusters.

### 3.2. GO Functional Annotation Analysis of Differentially Expressed Genes at Different Temperatures

**Figure 3:**
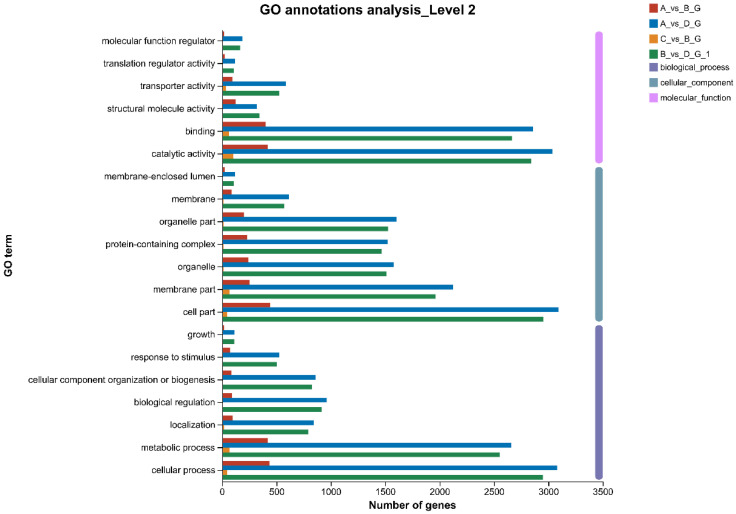
GO annotations shown for 20 GO terms of biological processes (BPs), cellular components (CCs), and molecular functions (MFs) at level 2 in *Tausonia pullulans* 6A7. The x-axis indicates the number of genes, and the y-axis indicates the GO term.

**Figure 4:**
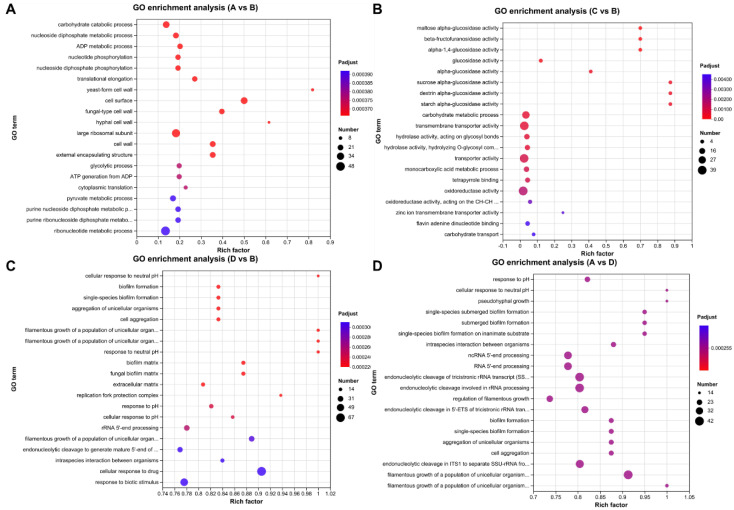
Bubble diagram of GO enrichment in *Tausonia pullulans* 6A7. (**A**) A vs. B GO enrichment. (**B**) C vs. B GO enrichment. (**C**) D vs. B GO enrichment. (**D**) A vs. D GO enrichment. The x-axis indicates the enrichment factor (the ratio of the number of genes enriched for the GO term to the number of annotated background genes; the larger the enrichment factor, the greater the degree of enrichment); the y-axis indicates the GO term.

GO is a database created by the Gene Ontology Consortium, and the GO database is used to classify biological processes in which genes are involved, the components that make up a cell, and the molecular functions that genes fulfill. At the time of the GO functional annotation, we found that 8455 DEGs were annotated with 1158 GO entries; of the 381 GO entries that were annotated between A and D, 295 entries were biological processes (BPs), 65 entries were cellular components (CCs), and 21 entries were molecular functions (MFs). We also found the most annotated entries for differentially expressed genes between groups A and D and between groups B and D (Figure 3). We selected 20 GO-enriched terms to draw a GO-enriched bubble diagram and analyzed the GO enrichment in the different groups (Figure 4). Between groups A and B, 104 GO entries were significantly enriched, including 70 BPs, 24 CCs, and 10 MFs (Figure 4A); between groups C and B, 15 GO entries were significantly enriched, including 2 BPs and 13 MFs (Figure 4B); between groups D and B, 199 GO entries were significantly enriched, including 166 BPs, 28 CCs, and 5 MFs (Figure 4C); between groups A and D, 183 GO entries were significantly enriched, including 153 BPs, 24 CCs, and 6 MFs (Figure 4D). The GO enrichment results showed that the differentially expressed genes had different functions at different temperatures; the same genes had different functions at different temperatures.

### 3.3. KEGG Annotation Analysis of Differentially Expressed Genes at Different Temperatures

**Figure 5:**
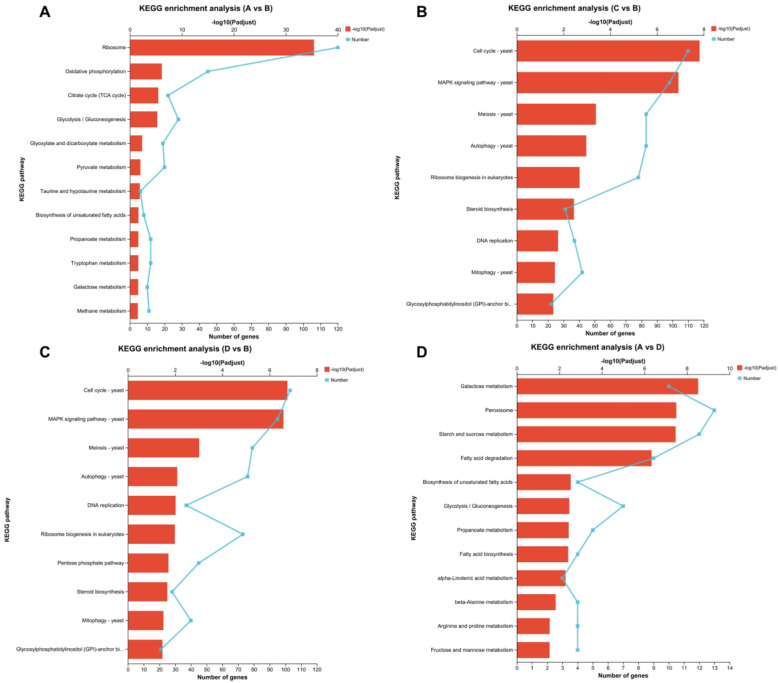
KEGG enrichment analysis of DEGs in *Tausonia pullulans* 6A7. (**A**) Line graph of the KEGG enrichment analysis of A vs. B with bars. (**B**) Line graph of the KEGG enrichment analysis of C vs. B with bars. (**C**) Line graph of the KEGG enrichment analysis of D vs. B with bars. (**D**) Line graph of the KEGG enrichment analysis of A vs. D with bars. The y-axis denotes the KEGG pathway; the upper x-axis denotes the number of genes on the pathway with the ratio corresponding to different points on the fold; the lower x-axis denotes the significance level of enrichment, which corresponds to the height of the bar, where the smaller the FDR, the larger the value of −log10 (FDR), and the more significantly enriched the KEGG pathway.

The KEGG annotation and enrichment analysis revealed and showed that between groups A and B, DEGs were significantly enriched in 12 KEGG pathways, which included those of ribosomes, oxidative phosphorylation, glycolysis/gluconeogenesis, the citrate cycle (TCA cycle), pyruvate metabolism, glyoxylate and dicarboxylate metabolism, propanoate metabolism, tryptophan metabolism, methane metabolism, galactose metabolism, the biosynthesis of unsaturated fatty acids, and taurine and hypotaurine metabolism (Figure 5A). Between groups C and B, DEGs were significantly enriched in nine pathways, namely, those of the cell cycle, MAPK signaling, meiosis, autophagy, ribosome biogenesis in eukaryotes, steroid biosynthesis, DNA replication, mitophagy, and glycosylphosphatidylinositol (GPI)-anchor biosynthesis (Figure 5B). Between groups D and B, DEGs were significantly enriched in 10 pathways, namely, those of the cell cycle, MAPK signaling, meiosis, autophagy, DNA replication, ribosome biogenesis in eukaryotes, pentose phosphate, Steroid biosynthesis, mitophagy, and glycosylphosphatidylinositol (GPI)-anchor biosynthesis (Figure 5C). Between groups A and D, DEGs were significantly enriched in 12 pathways, namely, those of galactose metabolism, peroxisomes, starch and sucrose metabolism, fatty acid degradation, the biosynthesis of unsaturated fatty acids, glycolysis/gluconeogenesis, propanoate metabolism, fatty acid biosynthesis, alpha-linolenic acid metabolism, beta-alanine metabolism, arginine and proline metabolism, and fructose and mannose metabolism (Figure 5D). Notably, between groups A and D, the pathways of fatty acid biosynthesis (map00061) and the biosynthesis of unsaturated fatty acids (map01040) were significantly enriched in the KEGG analysis. A vs. D will be the focus of our subsequent studies, as this comparison provided us with an important database for screening candidate genes in *Tausonia pullulans* 6A7.

### 3.4. Proposed Temporal Analysis of Differentially Expressed Genes at Four Temperatures

**Figure 6:**
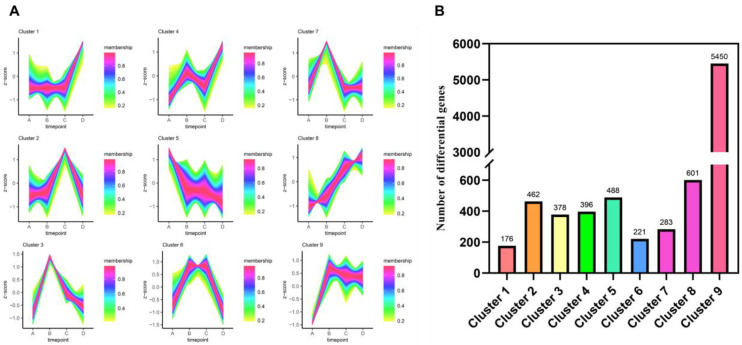
Proposed temporal analysis of 8455 differentially expressed genes in *Tausonia pullulans* 6A7 at four temperatures. (**A**) Proposed temporal analysis of DEGs in nine clusters between A and D. A is 15 °C, B is 20 °C, C is 20 °C (without corn oil), and D is 25 °C. (**B**) The number of differentially expressed genes in nine different clusters.

In the proposed temporal analysis of DEGs at four temperatures, we found 8455 differential genes that were assigned to nine clusters with different expression trends (Figure 6A). As shown in Figure 6B, the number of genes in different temporal clusters was counted, and it was found that the greatest number of DEGs were in cluster 9, with 5450 DEGs having the same expression pattern at the four temperatures; cluster 1 had the lowest number of differentially expressed genes, with 176 differentially expressed genes having similar expression trends at the four temperatures.

### 3.5. Screening of FAD Candidate Genes in Tausonia pullulans 6A7

**Figure 7:**
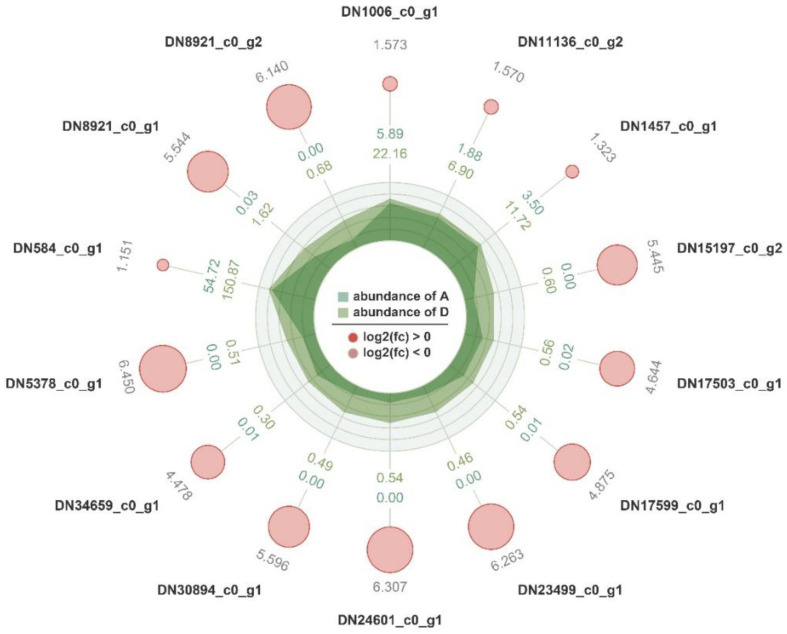
Screening of *FAD* candidate genes in *Tausonia pullulans* 6A7. The red circles indicate upregulated genes, and the size of the circle varies according to the size of the log_2_(FC) value. The dark green circles represent the average expression in sample A, the light green circles represent the average expression in sample D, and irregular shapes in the circles represent the expression abundance in samples A and D on each axis.

Between groups A and D, the pathways of fatty acid biosynthesis (map00061) and the biosynthesis of unsaturated fatty acids (map01040) were significantly enriched, so we screened and obtained 14 *FAD* candidate genes (Figure 7), all of which were upregulated at 25 °C in *Tausonia pullulans* 6A7. We also obtained two *FAD* (*DN29510_c0_g1* and *DN562_c1_g1*) genes that were expressed at all four temperatures. These gene sequences provide important candidate genes for the study of fatty acid production in yeast at different temperatures.

### 3.6. Gene Expression of FAD Candidate Genes in Tausonia pullulans 6A7 at Different Temperatures

**Figure 8:**
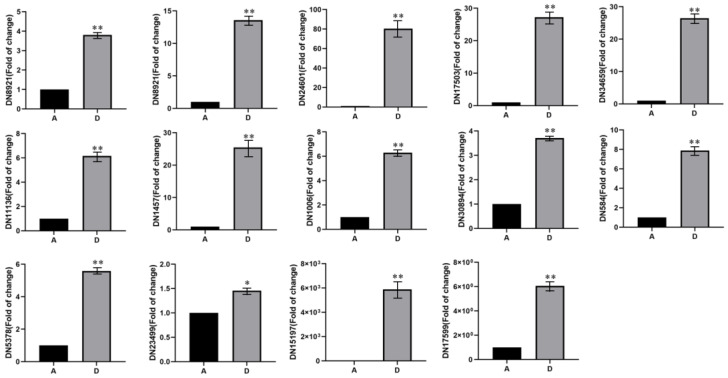
The expression of 14 *FAD* candidate genes for fatty acid biosynthesis in *Tausonia pullulans* 6A7 at temperatures A and D according to the fluorescent quantitative RT-PCR analysis. A is 15 °C and D is 25 °C. “*” indicates significance at *p* ≤ 0.05, and “**” indicates significance at *p* ≤ 0.01.

Based on the results of the KEGG enrichment analysis and GO annotation analysis, we screened and obtained 14 differentially expressed *FAD* candidate genes in the A vs. the D group. The gene sequences were named *DN8921_c0_g1*, *DN8921_c0_g2*, *DN24601_c0_g1*, *DN17503_c0_g1*, *DN34659_c0_g1*, *DN11136_c0_g2*, *DN1457_c0_g1*, *DN1006_c0_g1*, *DN30894_c0_g1*, *DN584_c0_g1*, *DN5378_c0_g1*, *DN23499_c0_g1*, *DN15197_c0_g2*, and *DN17599_c0_g1*. The fluorescent quantitative RT-PCR results (Figure 8) showed that these 14 differentially expressed *FAD* candidate genes had different expression patterns at different temperatures. The *FAD* candidate genes were more efficiently expressed at 25 °C, which was most likely due to fatty acid biosynthesis; these candidate genes can be used in yeast chassis cells for the targeted synthesis of certain fatty acids, and they can also provide a large number of important candidate genes for synthetic biology. In the meantime, the *DN15197_c0_g2* gene was expressed at 25 °C but not at 15 °C, and it was the only one of the 14 *FAD* candidate genes with this expression pattern that can be used for subsequent functional studies of fatty acid production in *Tausonia pullulans* 6A7.

**Figure 9:**
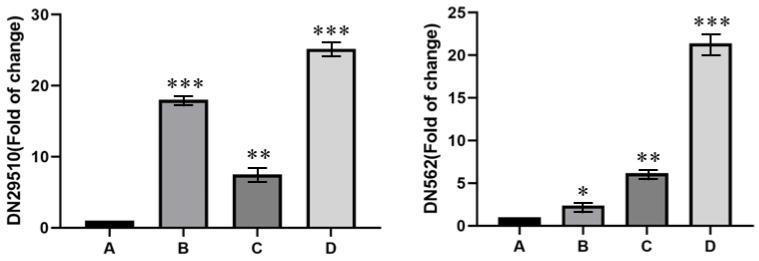
The expression of two *FAD* candidate genes (*DN29510_c0_g1* and *DN562_c1_g1*) for fatty acid biosynthesis in *Tausonia pullulans* 6A7 at all four temperatures according to the fluorescent quantitative RT-PCR analysis. A is 15 °C, B is 20 °C, C is 20 °C (without corn oil), and D is 25 °C. “*” indicates significance at *p* ≤ 0.05, “**” indicates significance at *p* ≤ 0.01, and “***” indicates significance at *p* ≤ 0.001.

We also screened and obtained two *FAD* candidate genes (*DN29510_c0_g1* and *DN562_c1_g1*) that were expressed at all four temperatures (Figure 9). *DN29510_c0_g1* and *DN562_c1_g1* were significantly more highly expressed at both 20 and 25 °C in comparison with 15 °C. Meanwhile, the highest expression was observed at 25 °C. These results demonstrate that the *FAD* candidate genes were expressed more efficiently at 25 °C. The use of these functional genes can be used to determine the appropriate culture temperature in *Tausonia pullulans* 6A7. Ultimately, we determined that the data analysis and screening methods for *FAD* candidate genes in 6A7 were accurate.

## 4. Discussion

*Tausonia pullulans* 6A7 is a low-temperature yeast strain that can produce lipases [7]. The chassis cells of yeast are important components of synthetic biology, and utilizing yeast cells for fatty acid production is a new direction for cell factories to find sustainable supply routes [23]. Here, we investigated the numbers and patterns of differentially expressed genes in *T. pullulans* 6A7 at different temperatures. The number of differentially expressed genes (DEGs) in A (15 °C) vs. D (25 °C) was more than that in B (20 °C) vs. D (25 °C). In addition, the numbers of differentially expressed genes in A vs. D and in B vs. D were significantly greater than the numbers of genes in both A (15 °C) vs. B (20 °C) and B (20 °C) vs. C (20 °C without corn oil). The 6A7 cells were cultured at a very low temperature (15 °C) at which they were in a state of recovery and growth and their metabolic activity was relatively slow, so their gene expression was lower at this temperature. However, 6A7 entered into an exponential growth period with the increase in the culture temperature, and it became more vigorous in life activity and had an increased metabolic level. When a culture temperature of 25 °C was reached, the metabolic and physiological activities were appropriate, the gene expression pattern was more active than that at low temperatures, and the number of differentially expressed genes was also higher. In this study, the culture temperature was shown to affect *T. pullulans* 6A7 as a chassis cell for the directed production of lipase or metabolism, as these are important indicators in yeast cell factories.

In order to further investigate the functions of our screening of differentially expressed genes (DEGs), it should be noted that these DEGs were mainly found to be involved in binding, catalytic activity, membrane parts, cell parts, metabolic processes, cellular processes, and other processes in the GO annotation. The significant enrichment of metabolic processes according to the GO annotation reflected the biological significance of yeast as a cell factory. Synthetic biology aimed at standardizing, modularizing, and innovating cellular functions has made great strides with the rapid advances in DNA synthesis and next-generation sequencing technologies [24]. Here, we review important advances in the synthetic biology of the brewer’s yeast *Saccharomyces cerevisiae*, an important eukaryotic model and widely used cell factory [25]. The KEGG annotation results revealed and showed that the DEGs were significantly enriched in the pathways of fatty acid biosynthesis and the biosynthesis of unsaturated fatty acids. Based on the GO annotation and KEGG enrichment analysis, *T. pullulans* 6A7 was shown to be a yeast strain that can produce lipases. This finding is consistent with previous results reported in the literature.

It has been shown that cold-active lipases exhibit high activity at low temperatures (0–20 °C), but they have reduced thermostability [26]. It has also been reported that the optimal temperature of the lipase of *T. pullulans* (40–50 °C) is very favorable [27]. This lipase is thermostable at 40–50 °C in *T. pullulans*, even though it is a cold-adapted yeast. However, we found the highest expression of the lipase-producing candidate gene at 25 °C in *T. pullulans* 6A7, which is where the enzyme may be at its most active. Unfortunately, we did not measure the enzyme activity at that temperature, and this will be the focus of our future research.

The members of the fatty acid desaturase (*FAD*) gene family have been mainly studied in many plants, such as in *Gossypium raimondii* [28], *Cucumis sativus* [5], *Linum usitatissimum* [29], *Musa* spp. [30], *Cicer arietinum* [31], *Camelina sativa* [32], etc., but they have not been systematically isolated and studied in *Tausonia pullulans*. In *Gossypium raimondii*, nineteen *FAD* genes were cloned at low temperatures, and their expression was investigated [28]. This also provided a theoretical basis for us to study *FAD* genes in *T. pullulans* 6A7 at low temperatures. Related studies have shown that the synthesis of fatty acids is regulated by a variety of genes [33]. *FAD* genes can encode enzymes that catalyze the desaturation of fatty acids, thus affecting their oxidative stability and nutritional value. The *FAD* genes were named after homologous genes in *Arabidopsis*. However, the *FAD7* and *FAD8* genes in *Arabidopsis* were highly homologous, and it was difficult to distinguish between them. However, it was shown that the *FAD7* gene was highly expressed at high temperatures, whereas the *FAD8* gene was highly expressed at low temperatures. In our study, 16 *FAD* candidate genes were screened and obtained from *G. pullulans* 6A7 at 25 °C. The *FAD* candidate genes were more efficiently expressed at 25 °C, which was most likely due to fatty acid desaturation. The *FAD* genes were expressed differently at both 15 °C and 25 °C. We only analyzed and compared the lowest and highest temperatures in this study, and from these two temperatures, we found 14 *FAD* genes to be more highly expressed at 25 °C, and we believe that the transcription of these *FAD* genes is temperature-dependent. The next step should be to analyze the functions and regulatory mechanisms of these *FAD* candidate genes in *T. pullulans* 6A7 and to use these genes for targeted fatty acid desaturation in yeast cell factories.

Fortunately, we also found two *FAD* candidate genes (*DN29510_c0_g1* and *DN562_c1_g1*) that were expressed at all four temperatures. The *DN29510_c0_g1* and *DN562_c1_g1* genes had slightly different expression patterns; *DN29510_c0_g1* was highly expressed at 20 °C and 25 °C, while *DN562_c1_g1* was highly expressed at 25 °C. The significant expression of *DN29510_c0_g1* and *DN562_c1_g1* genes can provide us with a direction for studying the function of *FAD* genes in *T. pullulans* 6A7. Meanwhile, our future studies will investigate these two *FAD* candidate genes for the detection of molecular markers of lipase-producing genes.

## Data Availability

The transcriptome data of *Tausonia pullulans* (A, B, C, and D) in this study were deposited in NCBI under accession numbers BioProject PRJNA1002343, BioSample SAMN36840777—SAMN36840788, and SRA SRR25520477—SRR25520488.
